# Supplementary figures and images for: Estimation of sodium consumption by novel formulas derived from random spot and 12-hour urine collection
Source: PLoS One. 2021 Dec 2;16(12):e0260408. doi: 10.1371/journal.pone.0260408 (PMC8639059; doi:10.1371/journal.pone.0260408)

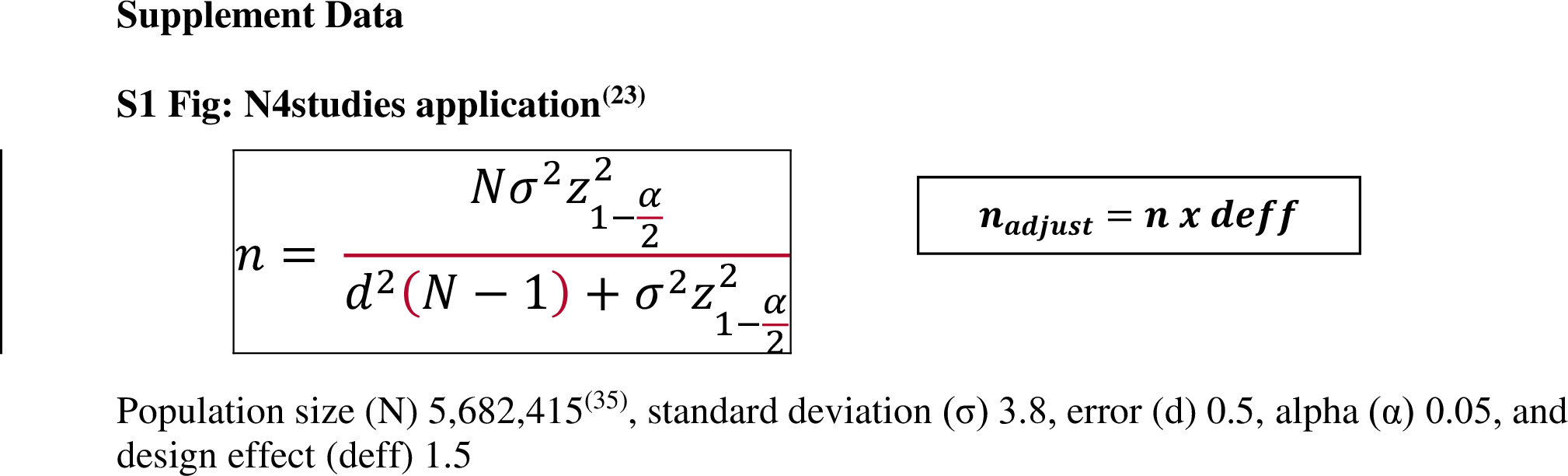

Supplement: S1 Fig — (TIF) [file pone.0260408.s001.tif]

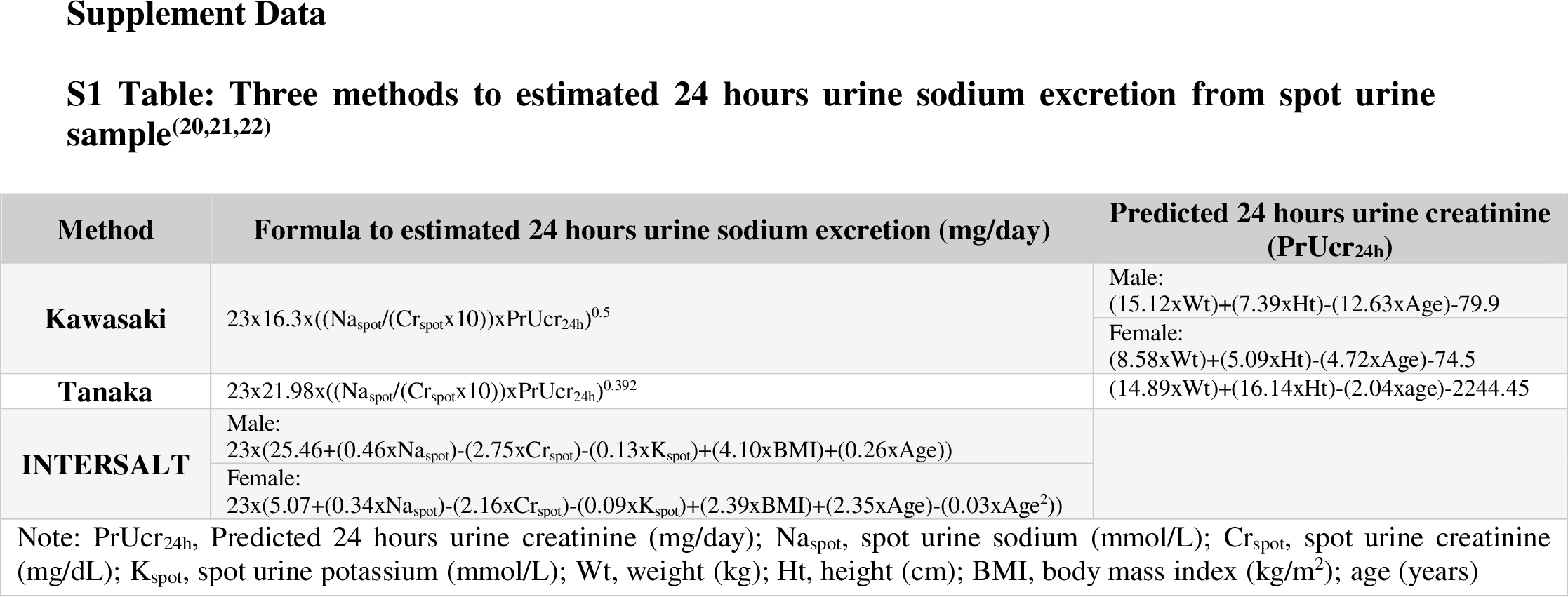

Supplement: S1 Table — (TIF) [file pone.0260408.s002.tif]

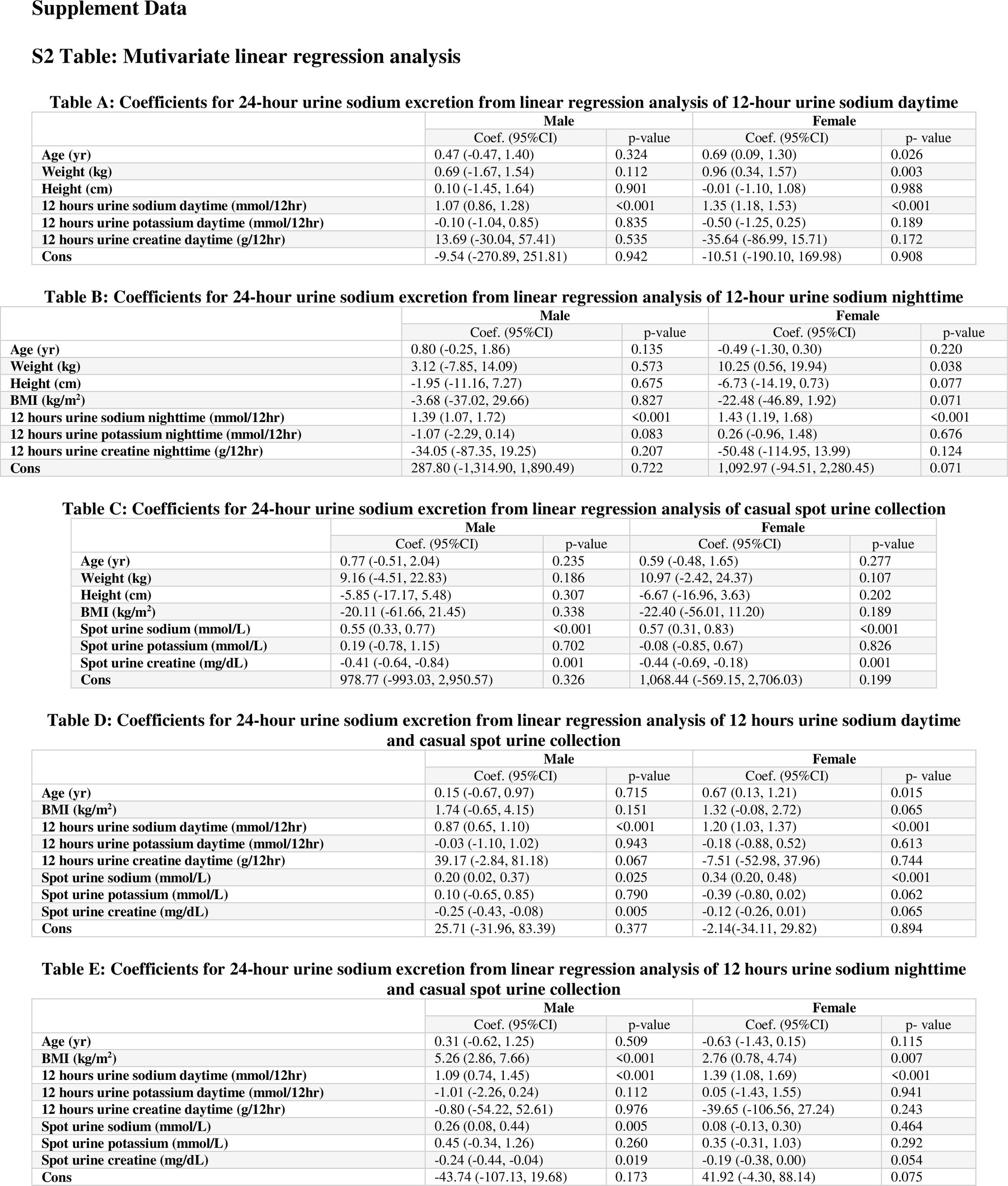

Supplement: S2 Table — (TIF) [file pone.0260408.s003.tif]
