# Supplementary material for: Estimation of sodium consumption by novel formulas derived from random spot and 12-hour urine collection
Source: PLoS One. 2021 Dec 2;16(12):e0260408. doi: 10.1371/journal.pone.0260408 (PMC8639059; doi:10.1371/journal.pone.0260408)
Supplement: Attachment — Submitted filename: Response to Reviewers.docx [file pone.0260408.s005.docx]

**Response to Reviewer**

- In the introduction the authors made a literature revision, but there were no reported differences between handling of sodium in the Western, Eastern and Black populations.

**Ans:** We have added the literature review in the introduction part as follows:

In 2010, the global mean sodium intake was 3.95 g/day (95% CI 3.89 to 4.01). This was nearly twice the WHO recommended limit of 2 g/day and equivalent to 10.06 (9.88–10.21) g/day of salt. Intakes were highest in East Asia, Central Asia and Eastern Europe (mean>4.2 g/day) and in Central Europe and Middle East/North Africa (3.9-4.2 g/day).^(5)^ In the UK and other developed nations, hypertension and its vascular complications were more common in ethnically African and South-Asian communities compared with Europeans.^(6,7)^ One important racial difference between ethnic groups is salt sensitivity and significantly suppressed activity of the renin–angiotensin–aldosterone system in African-origin hypertensive patients. As a consequence of this, they are more sensitive to a low-salt diet. There is also evidence that renin suppression is common in Japanese and Chinese hypertensive patients.^(8)^ As demonstrated in a systematic review, sodium reduction from a high sodium intake level (201 mmol/day) to a level of 66 mmol/day resulted in a decrease in SBP/DBP of 1/0 mmHg in white participants with normotension and a decrease in SBP/DBP of 5.5/2.9 mmHg in white participants with hypertension. A few studies showed that the effects in black and Asian populations were greater.^(9)^ (Paragraph2, Page3)

- In Methods is important to provide the calculation power performed for the inclusion of patients in the study.

**Ans:**

We have added a sentence describing the calculation of power in the statistical analysis section as follows: Power of test was assessed based on an expected minimum value of R squared of 0.6, sample size of at least 85, alpha error 0.05 and 6 predictors, the power of test was equal to 1.0. (Statistical analysis, Page7)

- The urine collection period was not described when the authors refer to the day or night period and also is important to know the start or end time of it.

**Ans:**

Regarding to urine collection period, the first morning void was discarded. A 12-hour urine daytime started from the second void and collected over a period of 12-hours, Then a 12-hour night time was collected until next morning including first morning urine. (Paragraph2, Page6)

- It is important describe the drugs that the volunteers normally received.

**Ans:**	We have added the information on drug used and discussed in the discussion part as follows:

In subgroup of participants with hypertension, numbers of patients receiving antihypertensive drugs were followings RAAS blockage (Azilsartan 1, Enalapril 8, Losartan 4), calcium channel blocker (Amlodipine 4, Manidipine 3, Lercanidipine 1), beta-blocker (carvedilol 1, propranolol 1), hydralazine in 1 and HCTZ in 1. (Paragraph2, Page13)

- The study analyzed other parameters in results as ages, gender, occupation, medical history, behavior towards dietary salt intake, hypertension and other parameters that were described in results, however, such analyzes were a little out of the objective of the study. The drugs received by patients need to be discussed with regard to the influence on the handling of sodium

**Ans:**

In participants with hypertension, some patients received antihypertensive drugs that may alter renal hemodynamics and sodium excretion. The administration of calcium-blocking drugs exerts a natriuretic response by exerting hemodynamic effects, as well as by acting directly on the proximal tubule and impairing sodium reabsorption in the distal tubule.^(30)^ In contrast, beta-adrenergic antagonists have little or no clinical effect on glomerular filtration rate (GFR), urinary sodium or potassium excretion, free water clearance, or body fluid composition.^(31)^ In animal models of salt sensitive hypertension, treatment with an angiotensin-converting-enzyme inhibitor (ACEI) or an angiotensin receptor blocker (ARB) effectively lowered blood pressure. In addition to lowering blood pressure, ACEI and ARB inhibition downregulated ENaC and suppressed sodium reabsorption in renal tubules.^(32)^ However, in our study, since the dosages of the antihypertensives were stable for at least 2 weeks before entering the study, the effect of medications on spot urine sodium excretion and in turn, estimated urine sodium excretion will be trivial as patients are in a steady state. Furthermore, a prior study showed that medications such as diuretics and ACE inhibitor or angiotensin receptor blocker did not substantially affect the accuracy with which Na excretion was estimated by spot urine equation.^(33^ (Paragraph2, Page13)

- Figures need to be better worked, with better resolution.

**Ans:**

We have revised the figures as suggested

- In the discussion, it would be important to emphasize the importance of the 12-hour urine collection findings in relation to the 24-hour urine in established calculations and its application in the future in the clinic.

**Ans:**

We added discussion on the importance of the new 12-hour urine equation and its application on page 14

- Native English proofreading is required

**Ans:**

Native English proofreading was performed
