# Supplementary material for: Estimation of sodium consumption by novel formulas derived from random spot and 12-hour urine collection
Source: PLoS One. 2021 Dec 2;16(12):e0260408. doi: 10.1371/journal.pone.0260408 (PMC8639059; doi:10.1371/journal.pone.0260408)
Supplement: Attachment — Submitted filename: Response to Reviewers.docx [file pone.0260408.s006.docx]

**Response to Reviewer**

1. The statistical analysis to Table 2 when the authors discuss age

Ans:

We have added ANOVA test was used for comparison of three means or more. (Paragraph1, Page7) and inserted P value in Table2.

2. In discussion explain the difference between HCW and non-HCW in excretions of sodium and creatinine.

Ans:

We have add discussion in Paragraph2, Page13

Furthermore, urine sodium excretion varied by age, with age groups of 30-44 and 45-59 having higher urine sodium excretion than the others. In line with our previous national surveys, sodium intake was higher among young people consuming higher calorie intake and fast food.^(14)^ Our study demonstrated that non-HCWs had significantly higher urine sodium excretion than HCWs suggesting more knowledge and awareness among HCWs leading to their lower salt intake. Studies have reported that in the general population, knowledge of the health impacts of high salt intake is low.^(30)^ Individuals with higher knowledge and awareness of the salt content and impact were significantly associated with lower salt intake.^(31,32)^ However, further research on the better understanding of salt knowledge and behavior in the population might facilitate the planning and implementation of a low salt intake program. Urine creatinine excretion in non-HCWs was also significantly higher than HCWs. This could be due to higher body mass index in the group of non-HCWs (26.05±5.12 kg/m2 in non-HCW vs 22.64±3.45 kg/m2 in HCW, P<0.001) which affected urine creatinine excretion.
